# Onwards and upwards: The development, piloting and validation of a new measure of academic tenacity- The Bolton Uni-Stride Scale (BUSS)

**DOI:** 10.1371/journal.pone.0235157

**Published:** 2020-07-23

**Authors:** Chathurika Sewwandi Kannangara, Rosie Elizabeth Allen, Jerome Francis Carson, Samia Zahraa Noor Khan, Gill Waugh, Kondal Reddy Kandadi

**Affiliations:** University of Bolton, Bolton, United Kingdom; Aalborg University, DENMARK

## Abstract

What factors determine success at University? For many years the construct of intelligence was felt to be critical. More recently, the construct of grit, has attracted the attention of many researchers, along with related concepts such as self-control, growth mind-sets and resilience. The authors of this paper have developed a specific measure of tenacity and self-composure, two constructs crucial to academic achievement. This measure comprises of 12 items drawn from the above constructs, but also including mental well-being and strengths use. In the first study, the authors report on the psychometric properties of the Bolton Uni-Stride Scale (BUSS). The new scale was administered to 1117 university students. Exploratory factor analysis (EFA) revealed two underlying factors, one labelled “tenacity” had seven items and accounted for 30% of the variance. The second was labelled “self-composure,” and accounted for 14% of the variance. In the second study the BUSS was given to 340 undergraduate students along with the Grit Scale, the Self-Control Scale, the Mind-sets Quiz, the Connor Davidson Resilience Scale (CD-RISC 10) and the short Warwick Edinburgh Mental Well-being Scale (WEMWBS). This study presented evidence for good internal consistency reliability (.74) and test-retest reliability over three weeks was .70 for Tenacity and .77 for Self-composure. BUSS Academic Tenacity correlated highly with grit (.63), self-control (.59), resilience (.52), mind-sets (.35) and mental well-being (.54). The study also evidences good discriminative validity of the BUSS. A second study conducted confirmatory factor analysis (CFA), explaining a total of 44% of the variance. The authors have shown good support for the reliability and validity of the BUSS scale. It now needs to be tested in other universities and in different countries. It is the contention of the authors that academic tenacity will be a better measure of academic success than other competing measures, such as grit, on their own. Further research is needed to test this assertion.

## Introduction

Identifying characteristics that are fundamental to academic achievement, apart from intelligence, is a worthwhile pursuit for educational research practice and institutions. Several factors and characteristics are strongly associated with academic achievement and success, such as grit [1]; resilience [2] strengths-use [3] self-control [4]; mind-set [5] and mental well-being [6].

### Grit

Of the above, the value of grit in academic achievement has attracted increasing attention from researchers [7–9]. The drive to persist in the face of adversity and to continue to persist in the pursuit of a long-term goal has been defined as “grit” and has been strongly associated with academic achievement [9]. If students express passion and perseverance towards long-term goals, they are more likely to express a higher level of academic achievement and achieve educational success [9]. Indeed, previous research has revealed that grit can be associated with academic productivity and engagement [10]; academic motivation [11]; academic achievement [1]; perseverance in challenging tasks [12]; academic performance [13]; amount of hours studying [14]; learning strategies [15]; task values and goal orientation [16]; the pursuit and attainment of postgraduate training [17] and the retention of students [18]. Additionally, research indicates that grit increases with age [19, 20]. Such that, previous research has highlighted an age-related trend of grit in university students, with older students demonstrating increased grittiness compared to younger students [21]. Although there is an age-related trend in the presence of grit, psychological research into grit has not demonstrated that there is a gender difference in the presence of grit [7, 9, 22]. The measurement of grit as an achievement-related characteristic is important for two reasons. First, it aids in determining which students are more likely to achieve and perform to a high standard, and those who are not. The second beneficial factor is the identification of students who present low levels of grit. This enables educators to direct them to support services that provide the opportunity for personal development and growth, further enhancing their grit, and leading to better academic achievement.

### Resilience

Educational research has demonstrated that some students will consistently perform poorly, while other students manage to overcome adversity and continue to thrive despite challenges or set-backs along the way [23]. Academic resilience is defined as the *“capacity to overcome acute and/or chronic adversity that is seen as a major threat to a student’s educational development”* [24]. Resilience has been shown to increase with age in high school students and in adulthood [25, 26]. However, it was posited that there were no significant age differences in resilience in university students as school leavers reported similar scores to mature students [27]. The concept of resilience in a university context is complex and the multidisciplinary nature of resilience is expected to impact this relationship with age [28]. It has also been consistently revealed that females are more resilient than males [29, 30]. Students who display this academic resilience choose to build on the challenges they face and perceive their previous misfortunes or failures as a motivator for future success. Academic resilience is also displayed in those students involved in facing and/or overcoming poverty [31] or abuse [32]. Resilience is often confused with grit as there is some overlap with their definitions and applications in educational and psychological research. Persistence, which is a core concept of grit, can predict academic resilience [23]. Nevertheless, it has been reported that resilience does not necessarily equate to grittiness, and vice versa [33]. It is therefore important to consider these two seemingly related characteristics as two separate measures that are related but distinct from one another. Likewise, much research has surfaced that illustrates academic resilience as a strong predictor of academic achievement [2, 34, 35]. Academic resilience has been further identified as a key aspect of student engagement and persistence on tasks [36]. Indeed, academic resilience is clearly a significant contributing factor towards students’ academic achievement and the measure of such a characteristic in students could greatly benefit the student population, as well as educators and institutions worldwide [37].

### Strengths-use

While research indicates that grit and resilience are important factors relating to academic achievement, it has also been highlighted that strengths-use is an important aspect that contributes to the academic achievement of students [3]. Focusing on weaknesses or adopting a deficit-based approach has several negative effects on students [38]. Indeed, focusing on the negative aspects of development and focusing on weaknesses reduces motivation, lowers confidence, demoralises students and lowers their expectations and effort exertion [38]. A strengths-based approach acknowledges that people experience challenges and difficulties, and identifies what students could achieve if provided with the appropriate and relevant support services. Research has revealed that by focusing on the development of strengths, instead of correcting flaws or weaknesses, generates more success in several personal development outcomes [39]. Additionally, focusing on improving strengths decreases levels of depression [40]. Through the identification of their strengths, students can explore their strengths and apply them in new and different ways. Research has demonstrated that understanding how one uses one’s strengths, how they have been used in the past and how they can be used in the future, enables the application of these strengths to different situations. This ultimately aids in the development of strengths-use and effects well-being, performance and achievement [41]. Therefore, enhancing students’ strengths-use has a direct effect on their academic achievement [42].

### Self-control

Self-control has been strongly associated with academic achievement, as research has suggested that students with high levels of self-control are likely to experience higher academic achievement [43]. Earlier research into this relationship established self-control as a vital contributor towards a student’s success in childhood [44–46]. Such that, Duckworth & Seligman [4], found that self-control was a better predictor of students’ Grade Point Average than intelligence. Likewise, the positive implications of self-control and self-regulation on academic success are evident in college and university students [47]. Indeed, a student’s ability to regulate their own study habits and control their impulses was significantly related to their final grades [48–50]. This implies that students’ success and academic achievement are dependent on their ability to self-regulate, monitor their behaviour and control their actions [4]. For the most part research demonstrates that levels of self-control are similar among female and male university students [51]; however some research has also demonstrated that self-control is particularly male dominated [52]. While there are some discrepancies in the gender differences in self-control, research has demonstrated that university students are faced with several difficulties which test their self-control. As age increases, so does the ability to exercise self-control [52, 53]. Yet, the relationship between self-control and university students remains unclear. Nonetheless, research has indicated that the natural occurrence of life events will encourage the development of resilience over time and research suggests that older adults have better emotional control [54]. Additionally, research shows the strong influence of self-control on academic achievement. A recent study revealed that students who express high levels of self-control have the ability to take control of their immediate environment and control their actions, for instance by switching off their phone to avoid being distracted [55, 56]. Also, it has recently been reported that students experience greater psychological distress than the general population [57]. Therefore, cultivating self-control in students may hugely benefit their overall well-being in addition to their academic achievement.

### Mind-set

Adopting a growth mind-set has been strongly associated with enhanced academic achievement [58, 59]. Individuals with a “growth mind-set” believe that their efforts and developments can change their abilities and qualities [58]. By changing the mind-sets of students and changing their perspectives on learning, mind-set interventions provide the tools to improve academic performance and achievement [60]. A study that asked underachieving students to participate in growth mind-sets and sense of purpose interventions, found that their grades improved significantly [60]. As we age, our mind-set evolves from a fixed mind-set to a growth mind-set [58, 61]. Perhaps as we age we become more self-aware and our confidence and self-efficacy grow, which contribute towards a mind-set that encourages improvement [61]. This was shown in a group of people between the ages of 25 and 61, with a gradual improvement in mind-set. While studies focused on primary school students reveal similar results, little research investigates the effect of age on mind-set in university students. It is worthwhile mentioning that a healthy sense of self-confidence predicts a growth mind-set. Furthermore, men start out adolescence with a higher level of self-confidence, but over their lifespan, their confidence tends to decline. In contrast, females generally experience a gradual and steady increase in self-confidence over the years. Therefore, research suggests that women are more likely to have a fixed mind-set in earlier years, but due to the significant shift in self-confidence, by the age of 60 women are more likely to have a growth mind-set compared to men [61]. Further expanding on the interrelated nature of these achievement-related characteristics, it has been revealed that adopting a growth mind-set can develop an individual’s level of grit, which also facilitates the pursuit of long-term goals and enhances academic achievement [62]. Likewise, research has indicated that a students’ sense of belonging is enhanced when the academic environment facilitates a growth mind-set [63]. Students who report feelings of a “great academic fit,” which can be cultivated through growth mind-set interventions, have increased levels of academic motivation, performance and achievement and are less likely to drop out [64]. Therefore, the development of a growth mind-set not only increases academic achievement, but has also been shown to increase grit and well-being, which are also strongly associated with academic achievement and retention [62, 65]. Consequently, measuring and monitoring students’ mind-sets may enable educators to maximise the potential for academic success through the development of a growth-mind-set.

### Well-being

Research has demonstrated that poor psychological well-being has negative effects on a student’s academic achievement and success [66]. For instance, it was found that students who experience depression consistently have lower academic motivation and achievement [67]. A number of studies show subjective well-being is predicted by age [68]. Indeed, research observations seem to suggest that although subjective well-being is constant over the lifespan, life satisfaction greatly differs between individuals based on the year they were born [69]. Some research suggests there is an inverted U-shape representation of life satisfaction through the life cycle with happiness and life satisfaction, peaking in mid-life [70]. The majority of research into subjective well-being shows that happiness and general life satisfaction do not differentiate greatly between genders [71]. However, there are several studies that provide evidence to suggest that females report slightly higher subjective well-being than males in certain countries, but lower subjective well-being in other countries [71]. It has been proposed that students’ well-being and positive emotions have a significant role to play in their academic motivation, performance and achievement [6]. A series of studies investigating the academic benefits of positive emotions directly highlighted the significant influence that well-being has on academic achievement. Findings showed that student’s positive emotions were related to increased motivation and effort, improved cognitive functioning, increased self-regulation and improved grades and exams scores [6]. It was therefore suggested that students who have an increased sense of well-being and enjoyment in learning are more likely to exert effort in their studies and as a result experience higher academic achievement. This directly reflects the importance of regulating and guiding student’s development of well-being.

### The interrelated nature of these distinct constructs

Although these constructs have been discussed separately, they are strongly interrelated. Indeed, the relationship between resilience and psychological well-being has been widely researched [72, 73]. This research demonstrates that university students lacking in resilience have a heightened chance of experiencing psychological distress [74, 75]. This research that resilience contributes positively towards an individual’s mental well-being [76–78]. More specifically, resilient university students are increasingly likely to maintain their mental health as they have the ability to quickly recover from difficult situations [79, 80]. Additionally, a model was proposed that identified self-management and emotional control as integral components of resilience development in university students [28], which advocates the interrelated nature of self-control and resilience. By explaining to students that resilience is a tangible concept, one that can be taught, a growth mind-set starts to develop [58]. A growth mind-set enables students to embrace failure and see it is a learning opportunity. By learning from their mistakes and taking on constructive criticism, students will be effectively displaying a tendency to delay gratification–which is the embodiment of self-control and is important in the development and maintenance of resilience [28]. Moreover, research suggests that resilience is closely associated with grit [21, 81]. For instance, setting relevant and meaningful goals that are aligned with their interests develops a student’s sense of motivation and commitment that compels them to persist in the face of adversity. Clearly, these constructs are related to some degree. For instance, improving student self-control will cultivate academic resilience, which will improve psychological well-being and chances of success at university. Acknowledging the complex and multidisciplinary nature of academic achievement, what constitutes it and how it can be improved, will encourage a more holistic and targeted approach to boosting educational success in university students. Yet, the measurement of one or two of these factors alone is overly simplistic and to present a clear understanding of the student population, academic achievement and educational success should be considered as a multifaceted construct with numerous contributors [82]. Indeed, it is essential to measure all of these characteristics in order to measure the academic tenacity of students, with the potential for ultimately improving several academic outcomes. The measurement of such characteristics is essential to a fundamental understanding of the student population and provides the opportunity to identify direct at-risk students for peer mentoring and support services that can aid in their personal development and growth. By identifying students at-risk, it is possible to build upon and foster certain characteristics that have, time and time again, been strongly associated with an increase in academic achievement and over-all well-being. In support, previous research advocates that there are a combination of components that are fundamental to cultivating a thriving and successful student population [83]. Such that, it was posited that thriving students are particularly gritty and resilient. They are somewhat optimistic, adopt a strengths-based approach towards their development, express high levels of self-control and harvest a growth mind-set [83]. Although this framework was originally developed in relation to working with people with dyslexia within a positive psychology context, its fundamental concepts can be directly applied to academic tenacity and demonstrate what characteristics are necessary in order to cultivate a successful student population. In addition to highlighting the interrelated nature of these characteristics, it reinforces the significance of each of these characteristics in the pursuit of student success.

### Other characteristics important to academic success

While it is essential to acknowledge the importance of these constructs, it does not suggest that these are the only constructs shown to influence achievement in university students. In fact, academic achievement in university students has been strongly associated with self-efficacy [37]; mindfulness [84]; a sense of belonging [85] and several others. Rather than including every construct that has been previously reported to effect academic achievement in university students for review, focusing on a select few could be more productive. Indeed, focusing on the grittiness of university students encompasses a variety of factors that are closely associated with grit. For instance, an individual’s grittiness can predict mindfulness [86, 87]; self-efficacy [88] and a sense of belonging [89]. While it is important to acknowledge self-efficacy as a concept related to academic achievement [37], the essence of self-efficacy in university students, that is relevant to academic tenacity, can be captured by an assessment of their grit and mind-set. For instance, the extent to which an individual is confident in their abilities can be partly attributed to their belief that their cognitive capacity is not fixed and can be developed. Furthermore, self-efficacious university students are increasingly likely to cope with challenges and achieve their identified goals. It can therefore be argued that measuring a university student’s grit and mind-set will generate an adequate idea of their self-efficacy. Therefore, by measuring grittiness in university students, you are acknowledging other relevant and influential factors related to academic achievement. Likewise, resilience [28], psychological well-being [90] and self-control [91] are comprised of numerous constituent parts that aid in their abilities to predict academic achievement. Ultimately, the combination of these factors (grit, resilience, self-control, well-being, strengths-use and mind-set) appear to productively and effectively predict academic achievement in university students. It is the combination of these constructs that equate to how tenacious a student will be in their academic studies. Collectively, these characteristics will promote tenacity and success in university and arguably in later life. Academic tenacity can therefore be defined as the combination of grit, resilience, strengths-use, self-control, psychological well-being and a growth mind-set that provides students with the capacity to thrive at university.

### Measuring academic tenacity

Due to the multidisciplinary nature of academic tenacity and its relationship with academic achievement, research in this area explores a multitude of contributing factors. Thus, it is not uncommon for a researcher to administer several assessments to their participants. For instance, one study looking at the relationship between grit, self-regulated learning and academic achievement involved the completion of 136-items [92]. Participation in multiple surveys takes time and effort and if participants are asked to complete too many, their motivation and attention will decrease [93]. Disengaged participants will then begin to produce perfunctory responses encouraging respondent fatigue and significantly affecting the quality of the data [93].

While there is no scale to measure academic tenacity; there is a newly devised measure of academic resilience designed to capture specific adaptive cognitive-affective and behavioural responses of university students to academic adversity [37]. While the Academic Resilience Scale (ARS-30) showed good internal and construct validity, its sole focus is on the students’ ability to bounce back from academic adversity. While this ability is important to the academic success of students’, it is not the only significant contributing factor. Indeed, grit, self-control, mind-set, strength-use and well-being have also shown to significantly predict academic success in university students. To attribute all academic success to the ability of overcoming academic adversity seems illogical and untenable, much like it does to ignore the presence of other influential characteristics.

### The present research

As of yet, there is no one tool devised to measure these characteristics respectively. Instead, a series of individual tools would need to be distributed to ascertain students’ academic tenacity and its constituent components. There are too many assessments involved to measure academic tenacity, so the development of one measure, that functions well, is necessary to improve the quality of research in this area. Henceforth, it seems plausible that the measurement of academic tenacity should be incorporated into one, shorter, and more concise scale that considers the influential and necessary characteristics related to academic achievement. Namely, strength-use, grit, resilience, self-control, mind-set and well-being. Further investigation into the relationship between these constructs and determining their impact on academic outcomes is a worthwhile pursuit. In doing so, higher education institutions can begin to promote a targeted approach to improving achievement. Correspondingly, the Bolton Uni-Stride Scale was developed. The Bolton Uni-Stride Scale (BUSS) is a 12-item scale measuring students’ “academic tenacity” and involves the measurement of students’ strengths-use, grit, resilience, self-control, well-being and mind-set. This could aid in the facilitation of interventions that are designed to cultivate academic tenacity in student populations.

## Study 1

This study investigated the validity of the BUSS. It aimed to determine the factor structure of the BUSS through exploratory factor analysis (EFA) using Principal Axis Factoring (PAF) as the method of factor extraction. This study also aimed to examine the internal consistency of the measure and determine individual differences in BUSS scores. Specifically, whether gender and age impacted an individual’s self-reported BUSS score.

### Method

#### Ethics statement

This study was carried out following the research ethical standards of the British Psychological Society (BPS) and the study was approved by the University of Bolton Ethics Committee.

#### Participants

A total of 1117 first-year students from the University of Bolton were recruited. Every student who started a course on September 2017 at the University of Bolton was asked to take part in this study. Participants age, gender and program were reported. Such that, 658 females were recruited (58.9%) and 450 males were recruited (40.3%). Participants younger than 20 (423); between 20–24 (324); between 25–29 (107); between 30–39 (119); between 40–49 (61) and aged above 50 (28) were recruited. (See Table 1 for full description of participants).

**Table 1:** Demographic characteristics of participant sample.

Demographic Characteristic		Number of Participants (N)	Percentage of Sample (%)
Gender	Female	658	58.8
	Male	450	40.2
	Missing	11	1.0
Age	Below 20	429	39.4
	20–24	336	30.0
	25–29	108	9.7
	30–39	126	11.3
	40–49	62	5.5
	Above 50	28	2.5

#### Materials

Along with the following survey, a brief outline of the task was provided, stating what would be expected of the participants, as well as a short explanation about what the study was investigating and the reasons for doing so. Finally, the participant information sheet outlined the participant’s ethical rights and identified that anonymity would be ensured. A consent form was issued to each participant whereby they had to confirm their understanding of the study and what was required of them, and their agreement to take part in the research. Certain ethical rights of the participants were also stated, such as the right “to withdraw at any stage up until the point that the questionnaire was handed back” and that all participants were to be over the age of 18.

*Bolton Uni-Stride Scale (BUSS)*. Each participant was asked to complete the Bolton Uni-Stride Scale (BUSS). BUSS is a scale that was developed by Faculty members of the Psychology Department at the University of Bolton. The 12-item scale measures “academic tenacity” through the inclusion of several related constructs (strengths-use, grit, resilience, self-control, well-being and mind-set). For a full breakdown of items in the BUSS, see Table 2.

**Table 2:** Item breakdown of BUSS.

Item No.	Item Statement	Characteristic Measured	Response Style	Sourced/Adapted From
1	I use my strengths in various situations	Strength-use	Positive	Deductive Method /Literature Review
2	I sometimes do things that feel good in the moment but later have regrets about it	Self-control	Negative	Self-Control Scale [94]
3	I consider myself to be persistent and hard working	Grit	Positive	Grit Scale [7]
4	I know my actions are wrong, but sometimes I cannot stop myself	Self-control	Negative	Self-Control Scale [94]
5	I am comfortable at times trying new ways of doing things	Mind-set	Positive	Mindset Quiz [95]
6	I consider myself as very capable in handling personal challenges	Resilience	Positive	Connor-Davidson Resilience Scale [96]
7	Using my personal strengths is a regular habit of mine	Strength-use	Positive	Deductive Method Literature Review
8	I set specific goals for my education and career but after a while decide on a new set	Grit	Negative	Grit Scale [7]
9	I find it easy to make decisions	Well-being	Positive	Warwick-Edinburgh Mental Well-being Scale [97]
10	I find it difficult to focus on one project for a long time	Grit	Negative	Grit Scale [7]
11	I am generally able to work out a way forward in life	Well-being	Positive	Warwick-Edinburgh Mental Well-being Scale [97]
12	I am always looking for ways to improve my talents and skills	Mind-set	Positive	Mindset Quiz [95]

Participants rate their response along a 5-point Likert scale from “not at all like me” (1) to “very much like me” (5). The BUSS is comprised of positively and negatively phrased items. The answering format remained consistent throughout the BUSS and negatively worded items were recoded when preparing for analysis to ensure each of the scale items weighted equally. In doing so, a high score indicates greater tenacity and self-composure. The majority (10) of the items included in the new “academic tenacity” scale were selected and reworded from already existing scales. Indeed, the 3 grit items were adapted from the grit scale [7]; the resilience item was taken from the Connor-Davidson Resilience Scale (CD-RISC 10) [96]; the self-control items were selected from the Self-Control Scale [94]; the mind-set items were adapted from the Mindset Quiz [95] and the well-being items were taken from the Warwick Edinburgh Mental Well-being Scale (WEMWBS) [97]. These particular items from each scale were selected for inclusion as they were the items that correlated highest to the overall score for that measure. For instance, the 3 grit items from the grit scale [7] that correlated highest with the total grit score were selected for inclusion. The imbalance of items to represent each construct is due to the development of a scale that is concerned with “academic tenacity”. For instance, grit is important in helping students persist on difficult tasks and seems most relevant to the topic of academic tenacity and so, is represented by 3 items in the BUSS. The remaining 2 items, measuring strength-use, were generated based on an extensive literature review [98, 99], which is the most commonly used deductive method of item generation [100]. All items, including those generated by authors, were subject to review by the expert opinions of psychology professors and researchers [101], which is a method widely used to analyse content validity [102]. In fact, when using new, adapted or previously unexamined scale items, it is recommended that items should be reviewed by a panel of experts [103]. A combination of deductive and inductive methods are consistent with the recommended strategy for the creation of a new measure [104].

#### Procedure

Participants were asked to read the information sheet and if willing to take part, sign the consent form. The consent form contained a section that asked participants demographic information such as age and gender. Then, participants were asked to complete the BUSS.

The BUSS was completed in class alongside a measure of numeracy and literacy that was administered to all new students.

### Results

#### Factor structure

The 12 items of the Bolton Uni-Stride Scale (BUSS) were subjected to Principal Axis Factoring (PAF) using Varimax rotation. Principal Axis Factoring (PAF) is the most commonly utilized method of extraction for EFA and is most appropriate for the aim of the study. That is, to identify the least number of factors accounting for the common variance of a set of variables [105] [106]. As such, the chosen method of EFA was PAF. A Varimax rotation was appropriate as the extracted factors correlated at .221 [105]. This suggested the extracted factors were not strongly correlated, requiring an orthogonal rotation method [105].

Preliminary checks found that the bivariate correlation matrix indicated a number of substantial correlations. Also, the Kaiser-Meyer-Olkin value was .85, which exceeds the recommended value of .6 [107] and Bartlett’s Test of Sphericity was significant at < .001, which is below the recommended value of .05 [108]. It was also revealed that multicollinearity was not an issue, as the determinant was above .001 [109]. All of the individual measures of sampling adequacy appeared to be over .6 and so no items were considered for candidates of removal from the scale [109]. Collectively, these checks suggest that the data was suitable for factors analysis.

The Kaiser Criterion states that factors with an initial eigenvalue above 1 should be extracted [110]. PAF revealed the presence of two factors with eigenvalues greater than 1 (3.661, 1.704), explaining 30.51% and 14.20% of the variance respectively. Thus two factors were presented for extraction, explaining a total of 44.71% of the variance.

Following PAF, any item with factor loadings greater than .40 were retained [111]. All the items loaded strongly on the two factors, reporting a value above >.0 [111]. Table 3 reveals factor loadings, with loadings of >.0 highlighted in bold [111].

**Table 3:** Results of principal axis factoring with varimax showing rotated factor loadings for the BUSS.

12 Items of the Bolton Uni Stride Scale (BUSS)	Factor Loadings	
	Factor 1	Factor 2
**Factor 1 (Tenacity Factor)**		
(7) using personal strengths is a regular habit of mine	**.706**	
(6)–consider myself as very capable in handling personal challenges	**.686**	
(11) generally able to move forward in life	**.638**	
(1) I use my strengths in various situations	**.594**	
(12) I am always looking for ways to improve my talents and skills	**.588**	
(3) Consider myself to be persistent and hard working	**.532**	
(9) I find it easy to make decisions	**.476**	
**Factor 2 (Self-Composure Factor)**		
(4) I know my actions are wrong, but I cannot stop myself		**.654**
(2) do things that feel good in the moment but later regret		**.636**
(10) find it difficult to focus on one project for a long time		**.451**
(8) I set goals, but after a while I decide on a new set of goals		.306
(5) Not comfortable at times trying new ways of doing things		.297
Eigenvalue	3.661	1.704
% Variance	30.51	14.20
Cronbach’s Alpha	.796	.595

On inspection of the pattern matrix, with factor loadings presented in Table 3, item 5 (.297) and item 8 (.306) revealed factor loadings which are considered to be low [112]. However, the significance of factor loadings depends on the sample size [113]. According to Stevens [112], for a sample size of 1000, the loadings should be greater than .162. As shown in Table 3, factor loadings are considerably higher. With a sample size of over 1000, it can be assumed that even in a very large sample, the significance of the loadings can be considered statistically meaningful [111]. Therefore, all items were retained.

Internal consistency estimates of > .80 were sought [114]. However, on inspection of Table 2, it appears that the internal consistency reliability for factor 2 is considerably lower, at .595. Table 4 presents the means scores and standard deviations for the two factors of the BUSS based on a sample of undergraduate university students (*n* = 1117). The distribution for self-composure scores were appropriately symmetric [115], reporting a skewness of -.138. Skewness and kurtosis for self-composure scores are both negative, indicating that the data are slightly left- skewed and peaked (leptokurtic) compared to a normal distribution. By applying the rule of thumb and dividing each value by its standard error, gives -1.86 for skewness and -.671 for kurtosis, both within the ±1.96 limits suggesting that the departure from normality is not too extreme [113]. On the other hand, the distribution for tenacity scores were moderately, negatively skewed at -.565 [115]. By applying the rule of thumb and dividing each value by its standard error, gives -7.63 for skewness and -4.85 for kurtosis, both out with the ±1.96 limits, suggesting that the departure from normality is somewhat extreme [113].

**Table 4:** Mean scores and standard deviations for each factor of the BUSS scale.

Factor	No. of Items	Theoretical Range	Mean Score	Std. Deviation	Skewness	Kurtosis
Factor 1	7	7–35	28.10	5.92	-.565	.723
Factor 2	5	5–25	16.41	5.52	-.138	-.100

The extracted two factor model appears to be have a reasonable goodness of fit. Such that, the interpretability of the rotated factor matrix is high. For instance, item clustering suggests that factor 1 (items 1, 3, 6, 7, 9, 11, 12) represents tenacity while factor 2 (items 2, 4, 5, 8, 10) represents self-composure. In addition, the two factor model can reproduce the original correlation matrix. Such that, the raw differences between the original correlations and the reproduced correlations, also referred to as the residuals, were small. As a rule of thumb, the majority of the residuals should be trivial with fewer than 60% reporting absolute values greater than .05 [109]. Analysis revealed that just 10% of the residuals are greater than .05. Therefore, we can argue that the two factor model has done a reasonable job at reproducing the original correlation matrix.

#### Individual differences

A one-way ANOVA demonstrated that there were no significant differences in tenacity (factor 1) scores between males (28.23) and females (27.98), *f*(1, 1078) = 1.380, p = .240 (*Eta Squared* = .001). However, there was a significant difference in self-composure (factor 2) scores between male (16.08) and female (16.61) students, *f*(1, 1078) = 6.665, p = < .05, (*Eta Squared* = .006). In addition, Pearson’s correlational analysis identified a linear relationship between age and tenacity (.235) and self-composure (.239) score at *p* < .001. Such that, findings reflected an age-related trend, as the mean tenacity and self-composure scores increased with age.

### Discussion

#### Factor structure

PAF was conducted to explore the factor structure of the BUSS. PAF is the most commonly utilized construct validity method as it is effective in identifying underlying latent variables of a measure by exploring relationships among observed variables [101, 116]. Consequently, two factors arose: factor 1 –which was interpreted as a ***tenacity*** factor–and factor 2 –which was interpreted as a ***self-composure*** factor. The tenacity factor accounted for 30.51% of the total variance and the self-composure factor accounted for 14.20% of the total variance. Combined, they accounted for 44.71% of the total variance in BUSS scores. The most important factor (*factor 1*) represented a tenacity tendency, which accounted for 30.51% of the total variance. The tenacity factor is comprised of seven items that represent resilience; the use of personal strengths; positivity; a growth mind-set; adaptability; persistence, confidence and open-mindedness. The subsequent factor (factor 2) represented a self-composure tendency and accounted for 14.20% of the total variance. This factor is made up of 5 items that represent a lack of self-control, a lack of identified goals, close mindedness, uncertainty and a fixed mind-set. On inspection of factor loadings and which items load on each factor, it becomes apparent that each item in factor 1 (tenacity) is positively worded and each item in factor 2 (self-composure) is negatively worded. Including positive and negative items in a survey reduces the likelihood of extreme response bias and acquiescence bias. In doing so, participants are forced to be attentive and provide a meaningful response rather than respondents going into “auto pilot” and agreeing to all statements [117]. The differentiation of factors based on the wording of the items could be due to the method effects associated with negatively worded items [118]. This suggests that respondents experiencing a higher prevalence of psychological distress are more likely to endorse negatively worded items [119].

#### Individual differences

Data analysis revealed the presence of an age-related trend in tenacity and self-composure, with scores increasing as age does. This corresponds to our previous knowledge that has surfaced from psychological research that shows a positive relationship between age and some of the achievement-related characteristics that are included in the BUSS scale–particularly grit, resilience, strength-use, self-control, well-being and mind-set. Indeed, previous research presents an age related shift in self-control [52, 53]; grit [19, 20]; resilience [25]; well-being [68, 69] and mind-set [61]. It is believed that these characteristics increase as we age for numerous reasons. Such that, older students are more likely to have overcome more challenges and setbacks in life, as well as having generally more life experiences to draw upon. Younger generation university students reported suffering difficulties with impulse regulation [55] and are more likely to have alcohol abuse problems or difficulties with diet control [120]. However, with age, the ability to self-regulate and monitor our impulses and behaviour is enhanced, possibly due to the way our brain develops as we get older [121]. The cultivation of a growth mind-set is increasingly more likely the older we are. This could be explained by an increase in self-awareness and confidence [61]. Finally, there are many possible explanations for the increase in well-being with age. Perhaps the positive relationship between well-being and age in university students is a consequence of the relationship between age and other factors, such as resilience and self-control. As older students are more likely to express self-control and resilience, they are increasingly likely to cope with the stressors and demands of university life which, in turn, will minimize psychological distress.

There were no significant gender differences in tenacity score. These research findings gain support from previous research that too, does not show any gender differences in related characteristics such as grit [7, 8, 22]; self-control [51] and well-being [71]. Comparatively, there was a significant difference in self-composure score based on the gender of participants. Indeed, female university students reported a higher self-composure score. As a lower score reflects a more positive outcome, these findings suggests that female students are more likely to report a lack of self-control, a lack of identified goals, close mindedness, uncertainty and a fixed mind-set. This can be supported by some research that does, in fact, suggest a gender difference in resilience [29, 30] and mind-set [61]. Subsequently, females have been proven to be more resilient than males, possibly due to several pieces of research that have demonstrated that females are increasingly more likely to experience more challenges in the workplace [122, 123]. Also, women are more likely to adopt a fixed mind-set in earlier years, but due to the significant shift in self-confidence, by the age of 60, women are more likely to have to a growth mind-set [61]. As the majority of university students were between 20 and 24 years old, research would suggest that these students are more likely to be experiencing challenges in academic and professional life, and are less self-confident.

## Study 2

Study 2 was conducted to further investigate the reliability and validity of the BUSS. More specifically, it aimed to replicate the established structure resulting from PAF in study 1. In study 2, a Confirmatory Factor Analysis (CFA) was conducted to test the formally devised model to determine whether the chosen factors could be empirically supported. Study 2 also investigated the convergent validity of the BUSS as it aimed to compare the new scale to some already existing scales that are popular in terms of measuring characteristics that are related to academic tenacity. Particularly, the Self-Control Scale [96]; WEMWBS [97]; CD-RISC 10 [95]; the Grit Scale [7] and the Mindset Quiz [94]. Additionally, the current research aimed to further examine the age and gender differences in tenacity and self-composure.

### Method

#### Ethics statement

This study was carried out following the research ethical standards of the British Psychological Society (BPS) and the study was approved by the University of Bolton Ethics Committee.

#### Participants

A total of 340 foundation, undergraduate and postgraduate students from the University of Bolton were recruited. (See Table 5 for the full description of participants).

**Table 5:** Demographic characteristics of participant sample.

Demographic Characteristic		Number of Participants (N)	Percentage of Sample (%)
Gender	Female	184	54.1
	Male	141	41.5
	missing	15	4.4
Age	16–21	153	45
	22–26	83	24.4
	27–30	28	8.2
	31 and above	63	18.5
	missing	13	3.8

#### Measures

*Bolton Uni-Stride Scale (BUSS)*. Participants were asked to complete the 12-item BUSS scale. A full description of this scale can be seen in the Method section of Study 1.

*Psychological well-being*. To measure participant’s mental well-being, the WEMWBS was used [97]. This is a 7-item scale that is scored in the form of a rating scale with 1 being “none of the time” and 5 being “all of the time”. Statements included “I’ve been feeling close to other people” and “I’ve been feeling optimistic about the future”. “A Cronbach's alpha estimate of .89 (student sample) and .91 (population sample) suggests some item redundancy in this scale. Reliability analysis using the student population sample from the present study showed a similar Cronbach’s alpha estimate of .89. As shown by [97], test-retest reliability at one week was reported to be high (.83).

*Resilience*. The Connor-Davidson Resilience Scale (CD-RISC 10) [96] was utilised for this study, which is a ten-item resilience scale that is scored in the form of a rating scale–from “not true at all” (1) to “true nearly all the time” (10). For instance, statements included “I am not easily discouraged by failure” and “having to cope with stress can make me stronger.” According to [124], the scale demonstrated good test-retest reliability (.90) over a two week period. Reliability analysis using the population sample of the present study found similar internal consistency (.89) as found by [124], who reported a Cronbach’s alpha estimate of .91.

*Grit scale*. A twelve-item Grit Scale that was developed by Duckworth, Peterson, Matthews and Kelly [7] was used for the purpose of this study. The scale is scored in the form of a rating scale, asking students to respond to twelve statements, grading their answer from 1–5 depending on how they felt about each statement. For example, item 7 states “I often set a goal but later choose to pursue a different one”—with 1 being “not like me at all” and 5 being “very much like me”. According to [9], the scale has high internal consistency, reporting a Cronbach’s alpha estimate of .85. Internal reliability analysis using the data from the present study revealed a Cronbach’s alpha estimate of .89.

*Self-control*. A 10 item Self-Control Scale (SCS), adapted from Tangney, Baumeister & Boone [94] was used for this study. Again, the scale is scored in the form of a rating scale with participants responding to ten statements from “not at all like me” (1) to “very much like me” (10). The scale included statements like “I get distracted easily” and “I’m good at resisting temptation”. According to [94], test-retest reliability was high at .89 for the Total SCS score and .87 for the SCS. Internal consistency estimates of reliability were high and the Cronbach’s alpha estimate for the Total Self-Control Scale was .89 [96]. Likewise, internal reliability analysis using the participant sample form the present study showed a Cronbach’s alpha estimate of .80. Thus, the scale appears to have good internal consistency.

*Mind-set*. A 20-item mind-set quiz that involves identifying the extent to which participants agree or disagree with several statements linked to a growth or fixed mind-set. Participants responded with “strongly agree”, “agree”, “disagree” or “strongly disagree”. Following the completion of all 20 items, participants’ scores were totalled, and ranged between 0 and 60. While this generates reliability issues, this mind-set quiz was most relevant to the central focus of the study. Indeed, the original mind-set questionnaire devised by Dweck [58] focused on intelligence in children and was not relevant to the specific context of academic achievement and tenacity in university students. Statements included “your intelligence is something very basic about you that you can’t change very much” and “the harder you work at something, the better you will be at it.” This mind-set quiz was created by Emily Diehl [95] based on Carol Dweck's growth mind-set work [58]. This scale can be accessed at http://www.classroom20.com/forum/topics/motivating-students-with. As yet, internal consistency and test-retest reliability of this measure is undetermined. Internal reliability analysis using the participant sample of the present study revealed that the Mind-set Quiz has good internal consistency, reporting a Cronbach’s alpha estimate of .74.

#### Procedure

Once the participants had read the information sheet and signed the consent form, they were asked to complete multiple surveys (BUSS; grit scale, resilience scale, self-control scale, well-being scale and mind-set quiz). Those participants who were recruited through lectures were introduced to the study before lectures began and those willing to participate were given 10–15 minutes to complete the study. Those participants recruited in the social areas of campus were approached between 9am and 5pm and likewise given 10–15 minutes. Participants were given sufficient time to complete all surveys and guidance where needed. On completion, participants were thanked for their participation in the study and all documents were collected from each person.

### Results

#### Factor analysis

In order to support the internal structure of the BUSS and further explore reliability and validity, CFA was conducted. The 12 items of the Bolton Uni-Stride Scale (BUSS) were subjected to CFA using SPSS version 23 and AMOS version 26. Similar to study 1, it was reported that the data were suitable for factor analysis as the Kaiser-Meyer-Olkin value was .805, which exceeds the recommended value of .6 [107]. Again, Bartlett’s Test of Sphericity was significant at .01, which is below the recommended value of .05 [108].

Table 6 reveals factor loadings after CFA was conducted, showing two latent variables, with loadings of >.40 highlighted in bold [111]. All of the items loaded strongly on each factor as factor loadings are above 0.4 [111] as recommended by Stevens [111], except for item 5 (.361) loading onto factor 2 (self-composure). Nonetheless, all are statistically significant (*p* < .01).

**Table 6:** Maximum likelihood estimates of the oblique (promax) rotated factor loadings for the BUSS.

12 Items of the Bolton Uni-Stride Scale (BUSS)	Factor Loadings	
	Factor 1	Factor 2
**Factor 1 (Tenacity Factor)**		
(6) capable in handling personal challenges	**.763**	
(7) using personal strengths is a regular habit	**.746**	
(11) generally able to move forward in life	**.683**	
(1) use my strengths in various situations	**.637**	
(12) always looking for ways to improve talents and skills	**.568**	
(3) persistent and hard working	**.497**	
(9) find it easy to make decisions	**.492**	
**Factor 2 (Self-Composure Factor)**		
(2) do things that feel good in the moment but later regret		**.656**
(4) I cannot stop my actions		**.620**
(10) find it difficult to focus on one project for a long time		**.547**
(8) I set goals, but after a while I decide on a new set of goals		**.403**
(5) not comfortable trying new ways of doing things		**.361**

Self-composure and tenacity exhibited significant covariation (.108) at *p* < .01. The model is an acceptable fit to the sample data based on commonly accepted thresholds (χ2 = 174.1, df = 53, *p* < .01, CFI = .87, TLI = .84, RMSEA = .08). The χ2 is statistically significant which may indicate a lack of fit. However, the χ2 is impacted by sample size and given that SEM techniques such as CFA tend to be large sample techniques, it is not uncommon for the chi-square test to be statistically significant [125]. While the absolute measure of fit (chi-square) does not indicate a good model fit; incremental measures of fit (CFI, TLI) are indicating that there is an adequate fit [126]. However, the RMSEA, a different absolute measure of fit is within the accepted threshold (< .10) and indicates adequate fit at 0.08 [127]. Therefore, the results support the conclusions that the two latent factors (tenacity and self-composure) are relatively strong reflections of the associated observed variables.

#### Individual differences

Similar to study 1, a one-way ANOVA demonstrated that the tenacity score did not significantly differentiate between male participants (26.72) and female participants (26.46), *f*(1, 318) = .257, p = .257 (*Eta Squared* = .001). Unlike study 1, which demonstrated a significant gender difference in reported self-composure scores, study 2 found no significant differences, *f*(1, 318) = .819, p = .366, (*Eta Squared* = .003).

Likewise, Pearson’s correlational analysis identified a linear relationship between age and tenacity (.227) and self-composure (.227) score at *p* < .001. These findings concur with that of study 1, suggesting an age-related trend as the mean tenacity and self-composure scores increased with age.

#### Validity and reliability

Table 7 indicates that tenacity and self-composure positively correlate with each of the other scales that were utilised in this study. That is, both factors are positively correlated with the self-control scale [96]; the grit scale [7]; the CD-RISC 10 [95]; the WEMWBS [97] and the mind-set quiz [94]. This shows evidence of good convergent validity. Test-retest reliability was assessed at three weeks using Pearson correlation coefficients through a sample of 26 students. A test retest value of 0.70 at *p <* .*001* was obtained for Tenacity and 0.77 at *p <* .*001* was obtained for Self-composure for a period of 3 weeks.

**Table 7:** Correlation between tenacity, self-composure and other measures used.

Measure Taken	1	2	3	4	5	6	7
1.Tenacity	-						
2.Self Composure	.172**	-					
3.Self-Control	.417**	.549**	-				
4. Grit	.461**	.586**	.590**	-			
5.Resilience	.613**	.238**	.286**	.462**	-		
6.Well-being	.537**	.363**	.337*	.435**	.640**	-	
7.Mind-set	.278**	.339**	.223**	.347**	.310**	.258**	-

** Pearson Correlation is significant at the 0.01 level (2-tailed).

Correlational analysis indicated that the items of BUSS that were selected and adapted from respective scales are significantly correlated to the total score of the original scales (see Table 8). This suggests that the subscales of BUSS (grit, resilience, well-being, self-control and mind-set) do, in fact, measure the intended construct. On inspection of Table 8, the *r* value of Pearson Correlational analysis between the BUSS subscale and its respective scale are the highest among other correlations for that subscale. This is the case for all, except for resilience whereby the resilience scale (CD-RISC 10) has a slightly higher correlation with the well-being subscale of BUSS, at .503. Strength-use was not included in this analysis as the items for strength-use in BUSS were devised by the authors rather than adapted from an already existing scale.

**Table 8:** Correlational analysis between total BUSS subscale scores and total scores of respective scales.

	Grit (Grit Scale)	Resilience (CD-RISC 10)	Well-being (WEMWBS)	Self-control (SCS)	Mind-set (Mind-set Quiz)
Grit (BUSS)	.627**	.255**	.320**	.513**	.260**
Resilience (BUSS)	.327**	.474**	.412**	.320**	.243**
Well-being (BUSS)	.323**	.503**	.433**	.320**	.169**
Self-control (BUSS)	.493**	.213**	.323**	.545**	.310**
Mind-set (BUSS)	.354**	.400**	.416**	.292**	.326**

** Pearson Correlation is significant at the 0.01 level (2-tailed). Highlighted in bold is the r value for correlation between subscale of BUSS and respective scale.

### Discussion

#### Factor structure

In study 1, EFA, more specifically, PAF, was conducted to explore and identify the underlying latent variables of the BUSS. However, using EFA allows for more subjectivity in the decision-making process which is considered an issue in such statistical procedures [128]. To generate consistent results on the psychometric indices of any new scale, the combined use of EFA and CFA is recommended [104]. In CFA, the specific hypothesized factor structure that was identified in PAF, can be statistically evaluated. Therefore, study 2 conducted CFA to confirm the factor structure of the BUSS as proposed in study 1. Two factors, tenacity and self-composure, accounted for 44.52% of the total variance in BUSS scores. The same seven items loaded onto factor 1 (tenacity) as they did in study 1. Likewise, the same 5 items loaded onto factor 1 (self-composure) as they did in study 1. Such that, the estimated model fits the data which suggests that the factor structure replicates [100]. This research also follows the recommendation that data from different participant samples should be used for the EFA and CFA [100, 129], which ensures the testing of a hypothesises structure for a new data set [129].

The significance of factor loadings depends on the sample size [113]. According to Stevens [111], for a sample size of 1000, the loadings should be greater than .162. As shown in Table 3, factor loadings are considerably higher. With a sample size of over 1000, it can be assumed that even in a very large sample, the significance of the loadings can be considered statistically meaningful [111]. Likewise in study 2, for a sample of 300, loadings should be greater than .298 [113]. On inspection of Table 6, all loadings are greater than .470, again confirming that the loadings can be considered as statistically meaningful [111].

#### Reliability and validity

Following EFA in study 1, conclusions regarding the factor structure of the BUSS were restricted to the sample collected [113]. The factor structure found from PAF in study 1 was confirmed through a CFA in study 2. This suggests that using a different sample reveals the same factor structure [111] and assumes cross-validation of the BUSS factor structure, allowing for generalisation [111]. In addition, a large sample size ensures factor stability [104, 130]. For instance, ten participants to each item is widely considered the recommendation [131] whereas this study nearly tripled the recommended number of participants to ensure factor stability.

The current study demonstrated strong evidence to suggest good convergent validity of the BUSS. Indeed, students who scored high on tenacity and low on self-composure also scored high on self-control, grit, resilience, well-being and mind-set. Comparatively, students who scored low on tenacity and high on self-composure also scored low on self-control, grit, resilience, well-being and mind-set. Thus, tenacity and self-composure positively correlated to grit, self-control, well-being, resilience and mind-set. Henceforth, students who scored high on tenacity and low on self-composure demonstrated a habit of using their strengths and perseverance to pursue a long-term goal. These students also demonstrated the ability to overcome challenges and strive towards their goals despite adversity. They also demonstrated a high degree of self-control, a growth mind-set and an increased level of mental well-being. In support, previous research reinforces the close alignment between these factors and already existing knowledge regarding characteristics influencing academic achievement. Indeed, research has indicated that there is a strong relationship between academic achievement and grit [9, 21], resilience [2], strengths-use [3], self-control [43], mind-set [58, 59] and well-being [6]. As these are the core components that contribute towards academic achievement, it can therefore be proposed that the BUSS measures two key components (tenacity and self-composure) that are crucial to educational success.

Table 7 shows evidence of good convergent validity, demonstrating that tenacity and self-composure are strongly correlated with the other measures utilised in this study. Namely, the correlation between tenacity, self-composure, self-control, grit, resilience, well-being and mind-set are all significant at the 0.01 level (2-tailed). On inspection of Table 7, the slightly weaker correlation between tenacity and self-composure (.172) highlights the distinct nature between the two constructs, suggesting they are measuring different characteristics. While tenacity positively correlates with resilience (.613) and self-control (.417), there are clear differences in the strength of these correlations. This could be explained partly because tenacity is expected to be more highly correlated with the measures most related to its component parts. For example, items of the BUSS related to self-control load more strongly onto the self-composure factor than the tenacity factor. It is therefore unsurprising that the self-control scale more strongly correlates to the self-composure factor (.549) than the tenacity factor (.417). This is the same for the self-composure factor, which has a stronger correlation to those measures that best reflect its core characteristics. Internal consistency estimates of >.80 [114] were not achieved for the self-composure factor in study 1 (.595) or in study 2 (.651). This could be potentially explained by two reasons. First, the academic tenacity factor was represented by a greater disparity of items; while the self-composure factor was represented by only 5 items. Although this exceeds the recommendation of at least 3 items per factor [132], the 5 self-composure items are related to grit, self-control and mind-set. Whilst all 5 items are good indicators of self-composure, they represent three distinct constructs, which could potentially reduce the internal consistency reliability. Perhaps, the internal consistency reliability for self-composure was low as you can speculate that the items comprising this factor are generally “state” items rather than “trait” items. For example, one item in the self-composure factor reads “I find it difficult to focus on one project for a long time”, which more closely corresponds to a temporary set of feelings rather than a description of behaviours over a stable period of time.

#### Individual differences

Similar to study 1, the current study demonstrated an age-related trend in tenacity and self-composure, yet there were no significant differences depending on the gender of the students. This further reinforces the findings gained by study 1 and gains support from previous research (highlighted in study 1). Much like in study 1, there were no gender differences in tenacity score. However, contradictory to study 1 which revealed a significant gender difference in self-composure score, this study revealed no significant differences. The effect size in study 1 was relatively weak (*Eta Squared* = .001) and may only have been captured due to the large sample size. Study 2 had a smaller sample size and may not have been large enough to capture the effect of gender on self-composure score.

## General discussion

Academic achievement is reliant on a number of combined characteristics and in consideration of the continuing interest to study individual differences in academic achievement, there are very limited measures that encompass the most influential and desirable characteristics. Thus, the current research aimed to develop and validate a measure of tenacity and self-composure that considered these characteristics (grit, resilience, strengths-use, well-being, self-control and mind-set). The current research aimed to provide a valid measure of two separate constructs, tenacity and self-composure, that are relevant for research and practice in universities.

### Benefits and potential applications of the Bolton Uni-Stride Scale (BUSS)

The BUSS is the first measure of tenacity and self-composure that encompasses the measurement of several characteristics that have been closely associated with academic achievement. The BUSS provides a short and concise measure of tenacity and self-composure that can be utilised in different educational contexts to measure the academic tenacity of different student populations. In doing so, identification of languishing students (scoring low on self-composure and low on tenacity) provides educators with the opportunity to offer targeted intervention programs to improve academic achievement. Due to the potential relatability to the items of the BUSS and the generic wording that was used in their design, the BUSS can be applied to several educational contexts, including high schools and colleges. As such, the earlier tenacity and self-composure are instilled into the student population, the greater the benefit it offers students and educators. There are several potential applications of the BUSS. Indeed, the scale can be utilised to potentially cultivate tenacity and self-composure in different age groups and different educational environments.

### Limitations and future research

For both studies, it became clear that each item in factor 1 (tenacity) is positively worded and each item in factor 2 (self-composure) is negatively worded. Although, including positive and negative items in a survey reduces the likelihood of extreme response bias and acquiescence bias; the differentiation of factors based on the wording of the items could be due to the method effects associated with negatively worded items [118]. This suggests that respondents experiencing a higher prevalence of psychological distress are more likely to endorse negatively worded items [119]. It appears common in previous literature that reverse-scored items tend to be the worst fitting items in factor analyses and that factor structure can be split between a factor of straightforward worded items and a factor with reverse-scored items [133]. It is often stated that including reverse-coded items is necessary to avoid acquiescence among participants [100]. However, future research should consider choosing between avoiding acquiescence bias among participants by including a combination of positively and negative worded items; and preventing issues related to the use of reverse scores by not including items that are reverse-coded [100].

For both studies, internal consistency estimates of >.80 [114] were not achieved for the self-composure factor of BUSS. Indeed, Cronbach’s alpha estimates for study 1 (.595) and study 2 (.625) were below that of the minimal recommendation of .70 [101, 116]. The self-composure factor was represented by 5 items which exceeds the recommendation of at least 3 items per factor [132]. However, the 5 self-composure items are related to grit, self-control and mind-set, three distinct constructs. This could potentially reduce the internal consistency reliability of the comprising factor. Also, the items comprising the self-composure factor are generally “state” items rather than “trait” items, which more closely corresponds to a temporary set of feelings rather than a description of behaviours over a stable period of time. This could also implicate the internal consistency of the factor.

Contradictory findings were found regarding the effect of gender on self-composure scores. Indeed study 1 found a significant effect, and study 2 did not. Regardless, the effect size in study 1 was weak and may only have been captured due to the large sample size. Study 2 had a smaller sample size and may not have been large enough to capture the effect of gender on self-composure score.

The current research was only carried out at one higher education institution and the findings are therefore not generalizable to the general student population. Future research should consider utilising the BUSS at other educational institutions in order to provide a more holistic understanding that considers students of other ages, backgrounds and ethnicities. The present study revealed an underrepresentation of the male student population as the demographic statistics show a largely female participant sample. Further research should address the issue of generalizability of the BUSS to male students. According to UCAS in its annual publication of scheme-level data on admissions to full-time undergraduate higher education in the UK in 2015, there are more women than men in two thirds of university courses and further revealed that women are 35% more likely to attend university than males [134]. As with any research that uses questionnaires as means to gather data, there are some weaknesses. Along with the possibility or respondent fatigue [93], extreme response bias and acquiescence bias [118], social desirability stands to be an issue. Social desirability is a form of response bias whereby respondents to a questionnaire may over or under-report their behaviours to be viewed favourably by others [135]. The self-reporting nature of quantitative studies always raise the possibility of participant bias, social desirability, demand characteristics and response sets [136]. Future research could consider incorporating other objective or independent measure into their research to supplement the subjective evaluation of the items in the scale [100].

This research utilised cross-sectional methods of data collection, which inhibit the ability to determine a causal relationship. For instance, it remains unclear whether tenacity and self-composure contribute towards academic success, or comparatively whether academic success influences the development of tenacity and self-composure. Employing instruments at one point in time limits the ability to assess causal relationships [100]. Instead, future research could consider conducting a longitudinal study during scale development. This would generate a better understanding of tenacity and self-composure assess the predictive validity of BUSS [100].

Future research could also continue to explore the relationships among the tenacity and self-composure factors of the BUSS, and already existing measures related to academic achievement (grit, resilience, well-being, self-control and mind-sets). This could generate a clearer understanding of what constitutes towards tenacity and self-composure, the two key factors of BUSS.

## Conclusions

Previous research has indicated that the characteristics that are incorporated within the BUSS–that directly reflects the tenacity and self-composure of the student population—are crucial to academic achievement. While each factor correlates highly with the overall BUSS score, acknowledging the distinct nature of each factor could contribute towards targeted efforts to build tenacity and self-composure in university students. Ultimately, the BUSS directly measures the tenacity and self-composure of students and therefore provides a new measure of that is invaluable to educators and students. This then provides the opportunity to facilitate specifically designed intervention programs for students that are aimed at cultivating tenacity and self-composure, equating to greater academic achievement and educational success. The two studies reported in the present paper show that the new BUSS has strong psychometric properties and future research is needed to test this assertion.

## Supporting information

S1 File (ZIP)
